# CD147 reinforces [Ca^2+^]_i_ oscillations and promotes oncogenic progression in hepatocellular carcinoma

**DOI:** 10.18632/oncotarget.5225

**Published:** 2015-10-19

**Authors:** Juan Tang, Yun-Shan Guo, Xiao-Ling Yu, Wan Huang, Ming Zheng, Ying-Hui Zhou, Gang Nan, Jian-Chao Wang, Hai-Jiao Yang, Jing-Min Yu, Jian-Li Jiang, Zhi-Nan Chen

**Affiliations:** ^1^ Cell Engineering Research Center and Department of Cell Biology, State Key Laboratory of Cancer Biology, State Key Discipline of Cell Biology, Fourth Military Medical University, Xi'an, China

**Keywords:** hepatocellular carcinoma, CD147, [Ca^2+^]_i_ oscillations

## Abstract

Oscillations in intracellular Ca^2+^ concentrations ([Ca^2+^]_i_) mediate various cellular function. Although it is known that [Ca^2+^]_i_ oscillations are susceptible to dysregulation in tumors, the tumor-specific regulators of [Ca^2+^]_i_ oscillations are poorly characterized. We discovered that CD147 promotes hepatocellular carcinoma (HCC) metastasis and proliferation by enhancing the amplitude and frequency of [Ca^2+^]_i_ oscillations in HCC cells. CD147 activates two distinct signaling pathways to regulate [Ca^2+^]_i_ oscillations. By activating FAK-Src-IP3R1 signaling pathway, CD147 promotes Ca^2+^ release from endoplasmic reticulum (ER) and enhances the amplitude of [Ca^2+^]_i_ oscillations. Furthermore, CD147 accelerates ER Ca^2+^ refilling and enhances the frequency of [Ca^2+^]_i_ oscillations through activating CaMKP-PAK1-PP2A-PLB-SERCA signaling pathway. Besides, CD147-promoted ER Ca^2+^ release and refilling are tightly regulated by changing [Ca^2+^]_i_. CD147 may activate IP3R1 channel under low [Ca^2+^]_i_ conditions and CD147 may activate SERCA pump under high [Ca^2+^]_i_ conditions. CD147 deletion suppresses HCC tumorigenesis and increases the survival rate of liver-specific CD147 knockout mice by regulating [Ca^2+^]_i_ oscillations *in vivo*. Together, these results reveal that CD147 functions as a critical regulator of ER-dependent [Ca^2+^]_i_ oscillations to promote oncogenic progression in HCC.

## INTRODUCTION

Various cancer types differ significantly in terms of morphology, cell of origin, physiology, and pharmacology, but one thing common to most cancer cells is the requirement for intracellular Ca^2+^ signaling to maintain malignant phenotypes: sustaining proliferation, resisting cell death, enabling replicative immortality, inducing angiogenesis, and activating invasion and metastasis [1-4]. Ca^2+^-signaling pathways are remodeled or deregulated in cancer that result in changes in their physiology and distinguish them from non-malignant cells. Remodeling or deregulation of Ca^2+^-signaling pathways can provide means by which cancer cells can overcome systemic anticancer defense mechanisms [5-7]. Numerous studies have now established that some cancers are associated with major changes in the expression of specific Ca^2+^channels and pumps and that inhibition of some of these proteins inhibits the oncogenicity of cancer cells [8-10]. Although the dysregulation of Ca^2+^ signaling has been observed in tumor progression, the tumor-specific regulators of [Ca^2+^]_i_ oscillations are poorly characterized.

Depending upon the stimulus, Ca^2+^ signals are transmitted in the form of repetitive time-dependent changes in its concentration, known as [Ca^2+^]_i_ oscillations [11]. [Ca^2+^]_i_ oscillations are proposed to convey information in their amplitude and frequency, leading to the activation of specific downstream targets in response to sustained stimulation by extracellular signaling molecules [12,13]. The simplest mechanism proposed to describe [Ca^2+^]_i_ oscillations involves an increase in cytosolic Ca^2+^ (upstroke) due to the release of Ca^2+^ from intracellular stores following activation of extracellular Ca^2+^ influx; and a subsequent decrease in cytosolic Ca^2+^ (downstroke) occurs due to the refilling of the stores with Ca^2+^ following inactivation of extracellular Ca^2+^ influx [14,15].

As a cancer biomarker, CD147 is highly expressed in hepatocellular carcinoma (HCC) but weakly expressed in normal liver tissues[16]. CD147 is also significantly expressed in various cancers and appears to have prognostic significance, rendering it a possible cancer-associated biomarker for pathological diagnosis, prognostic evaluation,targeted therapy and radioimmunoimaging of a broad range of cancer types. [17] Prior studies have demonstrated that CD147 plays important roles in the progression of HCC, including migration, proliferation, tumor recurrence and poor prognosis [16,17]. Our prior study indicated that CD147 activates the FAK-PI3K-calcium (Ca^2+^) signaling pathway by interacting with a3b1 integrin and disrupts the NO/cGMP-mediated negative regulation of store-operated Ca^2+^ entry, thus increasing the intracellular level of Ca^2+^ level in HCC cells [18,19]. In this study, we demonstrated that CD147, functions as a critical regulator of ER-dependent [Ca^2+^]_i_ oscillations to correlate with the progression in HCC.

## RESULTS

### CD147 promotes HCC cells invasion, migration and proliferation by regulating [Ca^2+^]_i_ oscillations

**Figure 1:**
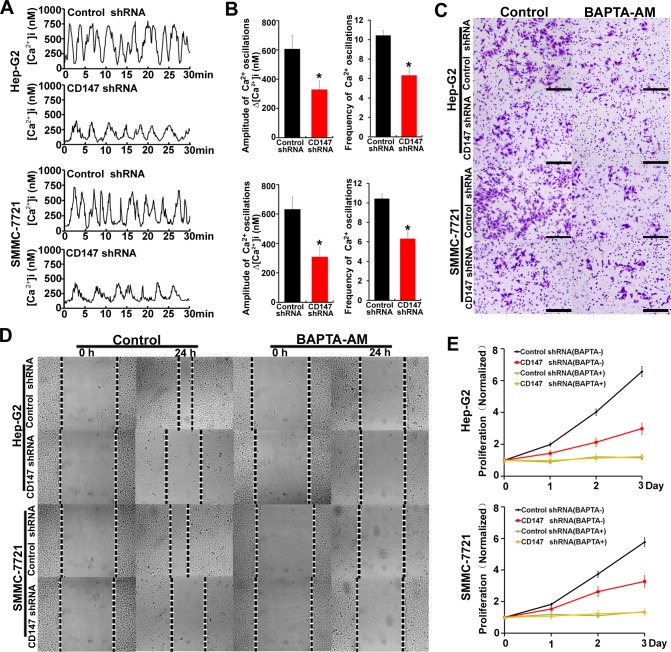
CD147 promotes HCC cells invasion, migration and proliferation by regulating [Ca^2+^]_i_ oscillations **A.** [Ca^2+^]_i_ oscillations were tested by [Ca^2+^]_i_ measurements in fluo-8-loaded control cells and CD147 knockdown cells after stimulation with EGF. **B.** Histograms of [Ca^2+^]_i_ oscillations amplitudes and frequencies in control cells and CD147 knockdown cells after EGF stimulation are shown. **C.** Invasive potential **D.** migration ability and **E.** normalized proliferation in control cells and CD147 knockdown cells incubated with or without BAPTA-AM. Bars represent each sample performed in triplicate, and the error bars represent the standard deviations. *p < 0.05, by Student's *t*-test.

We investigate the expression of CD147 in a range of hepatoma cancer-derived and nonmalignant liver cells. CD147 was highly upregulated in hepatoma cancer-derived cell lines (Supplementary Figure 1A). To assess the effect of CD147 on [Ca^2+^]_i_ oscillations, we used lentiviral delivery of shRNA constructs to knock down expression of endogenous CD147 proteins in HepG2 and SMMC-7721 cells (Supplementary Figure 1B-1E), and examined changes in [Ca^2+^]_i_ over time in individual cells, and examined changes in [Ca^2+^]_i_ over time in individual cells. [Ca^2+^]i oscillations were shown in both cells; interestingly, the amplitudes of the Ca^2+^ oscillations induced by EGF stimulation were lower in CD147 knockdown cells relative to control cells. In addition, CD147 knockdown cells displayed a lower frequency of [Ca^2+^]_i_ oscillations than control cells. Approximately 20% of responding control cells displayed ten [Ca^2+^]_i_ spikes or more, whereas CD147 knockdown cells responded with fewer than seven spikes over the same time period (Figure 1A & 1B). Together, these data suggested that CD147 modifies the nature of Ca^2+^ signals: CD147 knockdown triggers lower-amplitude and lower-frequency [Ca^2+^]_i_ oscillations in HCC cells, leading to an overall reduction in Ca^2+^ signal.

To detect whether the CD147-promoted cell oncogenicity was mediated by [Ca^2+^]_i_ oscillations in HCC cells, 1,2-bis (2-aminophenoxy) ethane-N, N, N’, N’-tetraacetate) (BAPTA-AM), an intracellular Ca^2+^ chelator, was used to block [Ca^2+^]_i_ oscillations. The results showed that CD147 knockdown markedly reduced cell invasion, migration and proliferation in HepG2 cells and SMMC-7721 cells. However, CD147 knockdown did not reduce cell oncogenicity following BAPTA-AM treatment (Figure 1C-1E). These results suggest that CD147 promotes HCC cell invasion, migration and proliferation by regulating [Ca^2+^]_i_ oscillations.

### CD147 contributes to ER Ca^2+^ release through FAK-Src pathway-mediated IP3R1 channel activation in human HCC cells

**Figure 2:**
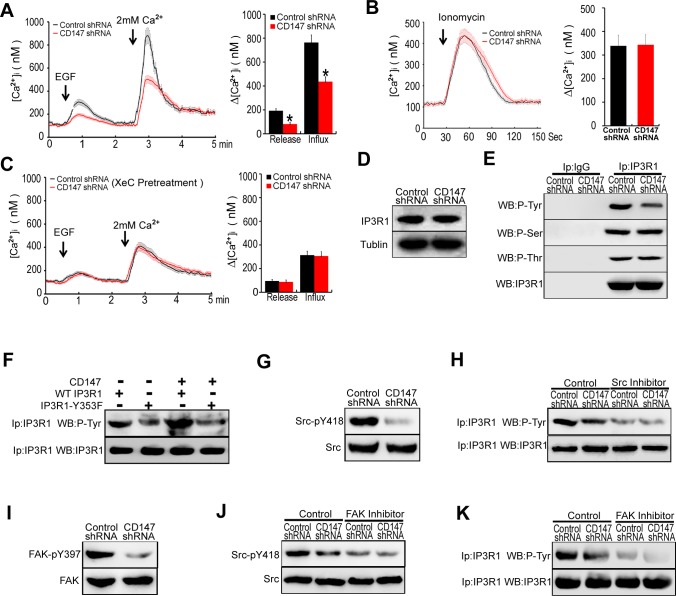
CD147 contributes to ER Ca^2+^ release through FAK-Src pathway-mediated IP3R1 channel activation in human HCC cells **A.** Average traces of [Ca^2+^]_i_ over time for cells stimulated with EGF in Ca^2+^-free medium were tested. Control cells, *n* = 18; CD147 knockdown cells, *n* = 14. **B.** Average [Ca^2+^]i traces following emptying of ER stores with 2 μM ionomycin for cells in Ca^2+^-free medium. Control cells, *n* = 8; CD147 knockdown cells, *n* = 11. **C.** Average traces of [Ca^2+^]_i_ over time for cells stimulated with EGF in Ca^2+^-free medium after IP3R inhibitor (XeC) treatment are shown. Control cells, *n* = 12; CD147 knockdown cells, *n* = 15. **D.** The expression levels of IP3R1 were examined. **E.** Cell lysates were immunoprecipitated with IP3R1 antibody and detected with a phospho-Tyr-specific antibody or a phospho-Ser-specific antibody or a phospho-Thr-specific antibody. **F.** Cell immunoprecipitates (IP) were analyzed with a general anti-phospho-Tyr antibody or IP3R1 antibody in cells expressing WT IP3R1 or IP3R1-Y353F mutant alone or in combination with CD147. **G.** The expression and phosphorylation levels of Src were examined. **H.** Analysis of phosphorylated Tyr in lysates from immunoprecipitates of IP3R1 in cells that were or were not pretreated with the Src inhibitor. **I.** The expression and phosphorylation levels of FAK were examined. **J.** Western blot analysis of phosphorylated Src in cells that were or were not pretreated with an FAK inhibitor. **K.** Analysis of phosphorylated Tyr in lysates from immunoprecipitates of IP3R1 in cells that were or were not pretreated with the FAK inhibitor. Bars represent each sample performed in triplicate, and the error bars represent the standard deviations. *p < 0.05 by Student's *t*-test.

Normally, [Ca^2+^]_i_ oscillations consist of two phases, an upstroke phase, contributed by ER Ca^2+^ release following the extracellular Ca^2+^ influx, and a downstroke phase, due to ER Ca^2+^ refilling. We next examined the roles of CD147 in ER Ca^2+^ release. EGF stimulation induced an increase in Ca^2+^ release in control cells, but this increase was minor in CD147 knockdown cells (Figure 2A). Upon addition of extracellular Ca^2+^, control cells displayed increased [Ca^2+^]_i_. This increase in [Ca^2+^]_i_ was blunted in CD147 knockdown cells (Figure 2A). Together, these data suggest that CD147 may increase Ca^2+^ release from ER stores and correspondingly increase the extracellular Ca^2+^ influx. To rule out the possibility of a difference in ER Ca^2+^ content, we measured the [Ca^2+^]_i_ of control cells and CD147 knockdown cells after treatment with ionomycin, which could transport calcium from the ER into the cytosol. In Ca^2+^-free medium, the peak levels of Ca^2+^ released by ionomycin were identical in control cells and CD147 knockdown cells (Figure 2B). The above results suggest that CD147 increases Ca^2+^ release from ER stores but does not alter the ER Ca^2+^ content.

IP3R is predominant ER Ca^2+^ releasing channel. We found that CD147 knockdown reduced Ca^2+^ release from ER stores. However, CD147 knockdown did not reduce Ca^2+^ release from ER stores following IP3R inhibitor Xestospongin C (XeC) treatment (Figure 2C). The result suggests that IP3R is involved in CD147-promoted Ca^2+^ release. Moreover, we found that the major IP3R isoform expressed in HCC is IP3R1 (Supplementary Figure 2A) and CD147 did not influence IP3R1 expression levels (Figure 2D). It is known that IP3R1 can be phosphorylated at tyrosine (Tyr), serine (Ser) and threonine (Thr). We then investigated the effects of CD147 on phosphorylated Tyr, Ser and Thr of IP3R1. The results showed that CD147 knockdown markedly decreased phosphorylated Tyr of IP3R1, however, CD147 knockdown did not change phosphorylated Ser and Thr of IP3R1 (Figure 2E). To directly address this issue, we constructed a Tyr353-mutated IP3R1 (IP3R1-Y353F). Immunoprecipitation (IP) showed that, compared with CD147 co-transfection with IP3R1 (WT), CD147 co-transfection with Tyr353-mutated IP3R1 (Y353F) suppressed IP3R1 Tyr353 phosphorylation levels (Figure 2F), indicating that CD147 may induce IP3R1 phosphorylation at Tyr353. To further detect whether the CD147-promoted cell oncogenicity was mediated by [Ca^2+^]_i_ oscillations in HCC cells, we tested the proliferation, invasion and migration in HepG2 cells which were transfected with IP3R1(WT) or IP3R1-Y353F vector. We found that CD147 promoted proliferation, invasion and migration in HepG2 cells transfected with IP3R1(WT) vector. However, CD147 did not apparently promote proliferation, invasion and migration in HepG2 cells transfected with IP3R1-Y353F vector (Supplementary Figure 2B-2D). The results strengthen the conclusion that CD147-related tumor behavior is [Ca^2+^]_i_ oscillation -related.

The tyrosine phosphorylation of IP3R1 is dependent on Src activity. We demonstrated that CD147 knockdown markedly decreased the Src phosphorylation in HepG2 cells (Figure 2G). Then we found that the Src inhibitor markedly decreased Tyr phosphorylation of IP3R1, and CD147 knockdown did not further reduce Tyr phosphorylation of IP3R1 after Src inhibitor treatment (Figure 2H). It is known that FAK activates Src through occupancy of the Src SH2 domain. We further confirmed that CD147 knockdown markedly decreased the FAK phosphorylation levels (Figure 2I). Moreover, FAK inhibitor significantly decreased the Src phosphorylation and Tyr phosphorylation of IP3R1, and CD147 knockdown did not further reduce Src phosphorylation and Tyr phosphorylation of IP3R1 following FAK inhibitor treatment (Figure 2G & 2K). These results suggested that CD147 promotes the Tyr phosphorylation of IP3R1 by activating FAK-Src pathway, thus inducing Ca^2+^ release from ER stores.

### CD147 contributes to ER Ca^2+^ refilling through CaMKP-PAK1-PP2A-PLB pathway mediated SERCA pump activation in human HCC cells

**Figure 3:**
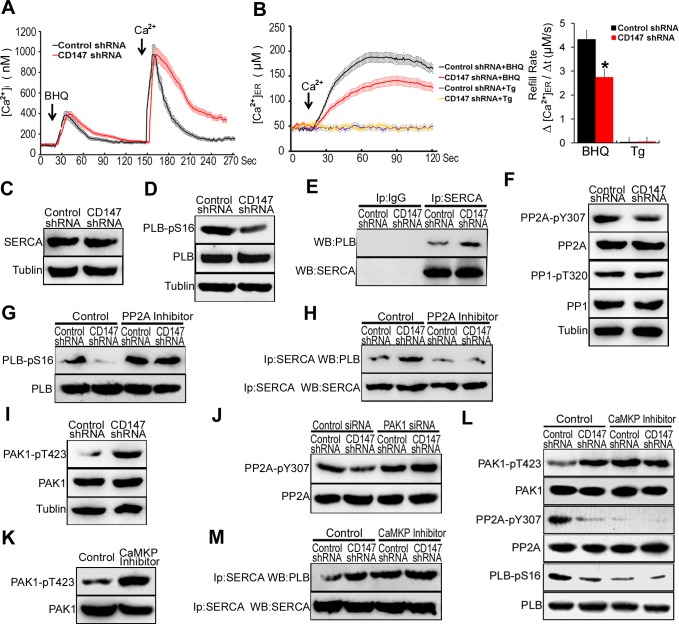
CD147 contributes to ER Ca^2+^ refilling through CaMKP-PAK1-PP2A-PLB pathway mediated SERCA pump activation in human HCC cells **A.** Average traces of [Ca^2+^]_i_ over time for cells stimulated with BHQ in Ca^2+^-free medium were shown and 2.5 min later Ca^2+^ (2.0 mM) was added. Control cells, *n* = 13; CD147 knockdown cells, *n* = 12. **B.** After cells were pretreated with BHQ or Tg to deplete ER Ca^2+^ store, we removed BHQ or Tg and added 2 mM Ca^2+^ to initiate Ca^2+^ refill. The [Ca^2+^]_ER_ was measured with mag-fura-2-AM. Control cells, *n* = 10; CD147 knockdown cells, *n* = 14. **C.** SERCA and **D.** phosphorylated PLB were tested. **E.** Endogenous SERCA complexes were isolated and examined for the presence of PLB by coimmunoprecipitation assay. IP with anti-lgG antibody was used as the negative control. **F.** Phosphorylated PP2A and PP1 were tested. **G.** Western blot analysis of phosphorylated PLB in cells after PP2A inhibitor treatment. **H.** Endogenous SERCA complexes were examined for the presence of PLB by coimmunoprecipitation assay after PP2A inhibitor treatment. **I.** Phosphorylated PAK1 were tested. **J.** Western blot analysis of phosphorylated PP2A in cells after PAK1 siRNA treatment. **K.** Western blot analysis of phosphorylated PAK1 in control cells and CaMKP inhibitor treated cells. **L.** Western blot analysis of phosphorylated PAK1, PP2A and PLB in cells after CaMKP inhibitor treatment. **M.** Endogenous SERCA complexes were examined for the presence of PLB by coimmunoprecipitation assay after CaMKP inhibitor treatment. Bars represent each sample performed in triplicate, and the error bars represent the standard deviations. *p < 0.05, by Student's *t*-test.

To assess whether CD147 regulates the downstroke of [Ca^2+^]_i_ oscillations, the ER stores of HCC cells were depleted of Ca^2+^ using BHQ (2,5-di-t-butyl-1,4-ben-zohydroquinone), which could lead to ER store depletion. After completely depleting Ca^2+^ from the ER, we removed BHQ and added 2mM Ca^2+^ to initiate ER Ca^2+^ refilling. Intriguingly, we found the downstroke of [Ca^2+^]_i_ oscillations in CD147 knockdown cells was slowed (Figure 3A). These results suggest that CD147 may accelerate the downstroke of [Ca^2+^]_i_ oscillations. It is known that the downstroke of [Ca^2+^]_i_ oscillations was influenced by the refilling of the ER stores with Ca^2+^ [20]. We measured the ER intraluminal Ca^2+^ concentration ([Ca^2+^]_ER_) with the low-affinity fluorescent Ca^2+^ indicator mag-fura-2 AM. We found that the refill rate was severely depressed in CD147 knockdown cells (Figure 3B). These results indicated that CD147 may accelerate ER Ca^2+^ refilling.

To address whether CD147-mediated Ca^2+^ refilling occurs through the SERCA pump, thapsigargin (Tg), an irreversible SERCA inhibitor, was used. The addition of Tg completely abolished CD147-mediated Ca^2+^ refilling, indicating that CD147-mediated Ca^2+^ refilling requires SERCA pump activity (Figure 3B). However, we found CD147 did not alter the expression of SERCA in HepG2 cells (Figure 3C). It has been well documented that phospholamban (PLB) negatively regulates SERCA activity in the ER by direct association. The phosphorylation of PLB at Ser16 causes its disassociation from SERCA, and the subsequent relief of SERCA inhibition [9]. After initiating ER Ca^2+^ refilling, we found that PLB Ser16 phosphorylation levels were markedly decreased in CD147 knockdown cells (Figure 3D). Corresponding co-IP tests further demonstrated that CD147 knockdown significantly increased the binding of PLB to SERCA (Figure 3E). These results demonstrated that CD147 causes PLB disassociation from SERCA and subsequent relief of SERCA inhibition by promoting the phosphorylation of PLB at Ser16.

Activated PP2A and PP1 could associate with PLB and dephosphorylation of PLB at Ser16. The phosphorylation of protein phosphatase1 (PP1) and phosphatase type-2A (PP2A) leads to inhibition of PP1 and PP2A enzyme activity, respectively [11]. The result that CD147 knockdown markedly increased the phosphorylated PP2A but did not influence the phosphorylated PP1 suggested that CD147 may suppress PP2A activity (Figure 3F). Moreover, CD147 knockdown did not further reduce phosphorylated PLB levels following PP2A inhibitor treatment (Figure 3G). Correspondingly, co-IP tests further demonstrated that CD147 knockdown did not increase the binding of PLB and SERCA following PP2A inhibitor treatment (Figure 3H). These results indicate that CD147 may promote PLB phosphorylation and enhance PLB dissociation from SERCA by suppressing PP2A activity.

The phosphorylation of p21-activated kinase 1 (PAK1) at Thr-423 induces a conformational change in PP2A that promotes PP2A dephosphorylation and increases its activity [11]. CD147 knockdown markedly increased the phosphorylation of Thr423 in PAK1 (Figure 3I). PAK1 siRNA led to a marked reduction of the PAK1 levels in HepG2 cells (Supplementary Figure 3A & 3B), and CD147 knockdown did not further increase PP2A phosphorylation following PAK1 depletion (Figure 3J). The result suggested that CD147 suppressed PP2A activity through inhibiting PAK1 phosphorylation.

Ca^2+^-dependent protein kinase phosphatase (CaMKP) associates with and dephosphorylates PAK1, leading to the inactivation of this kinase [21]. The CaMKP inhibitor treatment increased PAK1 phosphorylation in HepG2 cells (Figure 3K). Treatment of cells with CaMKP inhibitor blocked the CD147-induced dephosphorylation of PAK1 and CD147-induced phosphorylation of PP2A and PLB (Figure 3L). CD147 knockdown did not increase the binding of PLB to SERCA following CaMKP inhibitor treatment (Figure 3M). These data indicate that CD147 may inhibit PAK1 phosphorylation by activating CaMKP. Together, CD147 activates CaMKP to inhibit PAK1 phosphorylation and subsequently suppresses PP2A activity. CD147-inactivated PP2A increases phosphorylation of PLB and enhances disassociation of PLB from SERCA, thus promoting SERCA pump activity and rapid ER Ca^2+^ refilling.

### High [Ca^2+^]_i_ inhibits CD147-induced IP3R1 channel activation by antagonizing CD147-induced FAK phosphorylation

The above results demonstrated that CD147 contributes to both ER Ca^2+^ release and ER Ca^2+^ refilling in human HCC cells. To investigate how CD147 is able to achieve this biphasic effect, we tested the role of CD147 in low [Ca^2+^]_i_ conditions and high [Ca^2+^]_i_ conditions, as time-dependent changes in [Ca^2+^]_i_ concentration is the key feature of [Ca^2+^]_i_ oscillations. We generated low levels of [Ca^2+^]_i_ with BAPTA-AM, which is a membrane-permeable Ca^2+^ chelator, and generated high levels of [Ca^2+^]_i_ with Tg, which acts to irreversibly deplete ER Ca^2+^ stores and then activating a sustained Ca^2+^ influx.

**Figure 4:**
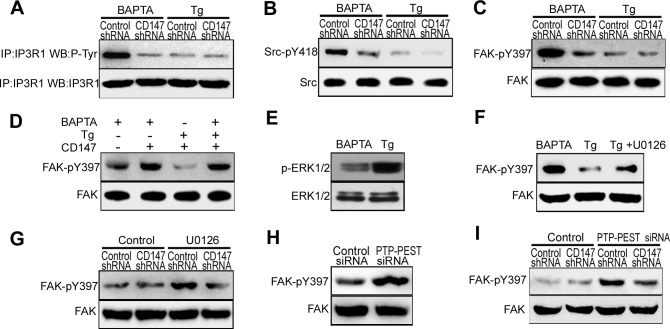
High [Ca^2+^]_i_ inhibits CD147-induced IP3R1 channel activation by antagonizing CD147-induced FAK phosphorylation **A.** Analysis of Tyr phosphorylation in lysates from immunoprecipitates of IP3R1 in cells that were pretreated with BAPTA-AM or Tg. **B.** Analysis of phosphorylated Src in cells that were pretreated with BAPTA-AM or Tg. **C.** Analysis of phosphorylated FAK in cells that were pretreated with BAPTA-AM or Tg. **D.** We generating low [Ca^2+^]_i_ conditions by 10μM the cell-permeant Ca^2+^ chelator BAPTA-AM and high [Ca^2+^]_i_ conditions by 4μM Tg and 2mM Ca^2+^. Phosphorylated FAK was tested in low or high [Ca^2+^]_i_ conditions. **E.** Analysis of phosphorylated ERK1/2 in cells that were treated with Tg or BAPTA-AM. **F.** Analysis of phosphorylated FAK in cells that were treated with Tg, or BAPTA-AM, or Tg and U0126 (ERK1/2 inhibitor) in combination. **G.** Western blot analysis of phosphorylated FAK in cells that were or were not treated with U0126. **H.** Western blot analysis of phosphorylated FAK in cells in which PTP-PEST was knocked down via siRNA. **I.** Western blot analysis of phosphorylated FAK in cells that were or were not treated with PTP-PEST siRNA.

We found that CD147 knockdown markedly decreased the tyrosine phosphorylation of IP3R1 in low [Ca^2+^]_i_ conditions, but had no effect in high [Ca^2+^]_i_ conditions (Figure 4A). Furthermore, CD147 knockdown markedly decreased phosphorylated Src and phosphorylated FAK levels in the absence but not in the presence of high [Ca^2+^]_i_ (Figure 4B & 4C). To further confirm these results, we examined FAK phosphorylation after a switch from low [Ca^2+^]_i_ to high [Ca^2+^]_i_. The level of phosphorylated FAK decreased after the culture medium was switched from low to high [Ca^2+^]_i_ (Figure 4D, lane 2,3) but returned to its initial high levels after Ca^2+^ was chelated by BAPTA-AM (Figure 4D, lane 4). These results demonstrate that the dephosphorylation of FAK by elevated Ca^2+^ antagonizes the effect of CD147 inducing FAK phosphorylation.

It has been well documented that Ca^2+^ can activate ERK1/2. Activated ERK1/2 may stimulate FAK dephosphorylation [12]. We found that phosphorylated ERK1/2 was less in low [Ca^2+^]_i_ conditions and increased in high [Ca^2+^]_i_ conditions (Figure 4E). The membrane-permeant ERK1/2 inhibitor, U0126 inhibited the dephosphorylation of FAK in high [Ca^2+^]_i_ conditions (Figure 4F). In addition, U0126 enabled CD147 to phosphorylate FAK in high [Ca^2+^]_i_ conditions (Figure 4G). Previous studies showed that ERK1/2 induces PTP-PEST phosphorylation. Isomerized PTP-PEST interacts with and dephosphorylates FAK at Y397. To assess whether PTP-PEST regulates the levels of FAK dephosphorylation, HepG2 cells were depleted of PTP-PEST with specific siRNA. Introduction of PTP-PEST specific siRNA in HepG2 cells resulted in marked reduction of PTP-PEST mRNA and protein (Supplementary Figure 4A & 4B). We found that the depletion of PTP-PEST was shown to increase FAK phosphorylation in HepG2 cells (Figure 4H). The phosphorylation of FAK induced by CD147 was inhibited in high [Ca^2+^]_i_ conditions, but PTP-PEST-specific siRNA inhibited the dephosphorylation of FAK in the presence of high [Ca^2+^]_i_ conditions and enabled CD147 to induce FAK phosphorylation (Figure 4I). These results demonstrate that in high [Ca^2+^]_i_ conditions, Ca^2+^-activated ERK1/2 promotes FAK dephosphorylation by PTP-PEST, thus antagonizing the phosphorylation of FAK by CD147 and blocking the activation of the IP3R1 channel by CD147. Collectively, these data show that CD147 may induce IP3R1 channel activation and increase Ca^2+^ release only under low [Ca^2+^]_i_ conditions.

### Low [Ca^2+^]_i_ inhibits CD147-induced SERCA pump activation by impairing CD147- induced CaMKP activation

**Figure 5:**
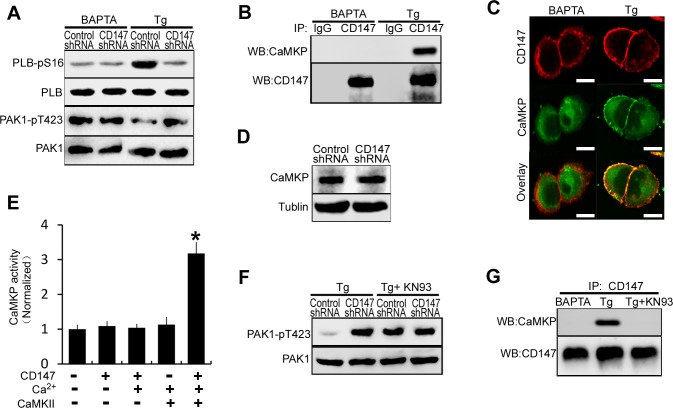
Low [Ca^2+^]_i_ inhibits CD147-induced SERCA pump activation by impairing CD147-induced CaMKP activation **A.** Western blot analysis of phosphorylated PLB and phosphorylated PAK1 in cells that were pretreated with BAPTA-AM or Tg. **B.** Endogenous CD147 complexes were examined for the presence of CaMKP by coimmunoprecipitation from cells that were treated with BAPTA-AM or Tg. **C.** The localizations of CD147 and CaMKP were determined by immunofluorescence staining in cells that were treated with BAPTA-AM or Tg (Bar = 10μm). **D.** Western blot analysis of CaMKP expression. **E.** CaMKP was incubated on ice for 30 min with the indicated proteins, and the phosphatase activities were determined with a solution-based assay using MUP as a substrate. **F.** Analysis of phosphorylated PAK1 in cells that were pretreated with Tg, or Tg and KN-93 (the CaMKII inhibitor) in combination. **G.** Endogenous CD147 complexes were examined for the presence of CaMKP by coimmunoprecipitation from cells that were treated with BAPTA-AM, or Tg, or Tg and KN-93 in combination. Bars represent each sample performed in triplicate, and the error bars represent the standard deviations. *p < 0.05 by Student's *t*-test.

To determine whether CD147 mediated ER Ca^2+^ refilling was affected by changing [Ca^2+^]_i_, the phosphorylation of PLB and PAK1 were examined in different [Ca^2+^]_i_ conditions. We found that CD147 knockdown markedly decreased the phosphorylation of PLB in the presence of high [Ca^2+^]_i_ but had no effect in the presence of low [Ca^2+^]_i_ (Figure 5A). Furthermore, CD147 knockdown markedly increased phosphorylated PAK1 levels in the presence but not in the absence of high [Ca^2+^]_i_ (Figure 5A). These results suggested that CD147 induces PAK1 dephosphorylation only under high [Ca^2+^]_i_ conditions. An analysis of CD147 complexes isolated from HepG2 cells confirmed that CD147 was co-immunoprecipitated with endogenous CaMKP under high [Ca^2+^]_i_ conditions but not low [Ca^2+^]_i_ conditions (Figure 5B). Immunofluorescent staining further showed the constitutive membrane colocalization of CD147 and CaMKP under high [Ca^2+^]_i_ conditions (Figure 5C). We then observed that CD147 knockdown did not alter the CaMKP expression levels (Figure 5D). CaMKP activity tests showed that CaMKP incubated with CD147 was not significantly activated in either the presence or the absence of Ca^2+^ (Figure 5E lane 2, 3). CaMKII, a Ca^2+^-active multifunctional protein kinase, which was found to phosphorylate and activate CaMKP [22,23], was added to the reaction mixture. CD147 increased CaMKP activation in the presence of CaMKII and Ca^2+^ (Figure 5E lane 4, 5). The dephosphorylation of PAK1 induced by CD147 in high [Ca^2+^]_i_ conditions was largely blocked by pretreatment with CaMKII inhibitor KN-93 (Figure 5F). In addition, the CD147-CaMKP interaction, which was found to be constitutive under high [Ca^2+^]_i_ conditions, was disrupted in cells treated with KN-93 (Figure 5G). These results suggested that CD147 interacts with and activates CaMKP in the presence of high [Ca^2+^]_i_ and that this interaction is mediated by Ca^2+^-activated CaMKII. Thus, CD147 may induce SERCA pump activation and the rapid refilling of the ER only under high [Ca^2+^]_i_ conditions.

### CD147 increases the amplitude of [Ca^2+^]_i_ oscillations through the FAK pathway and increases the frequency of [Ca^2+^]_i_ oscillations through the CaMKP pathway

**Figure 6:**
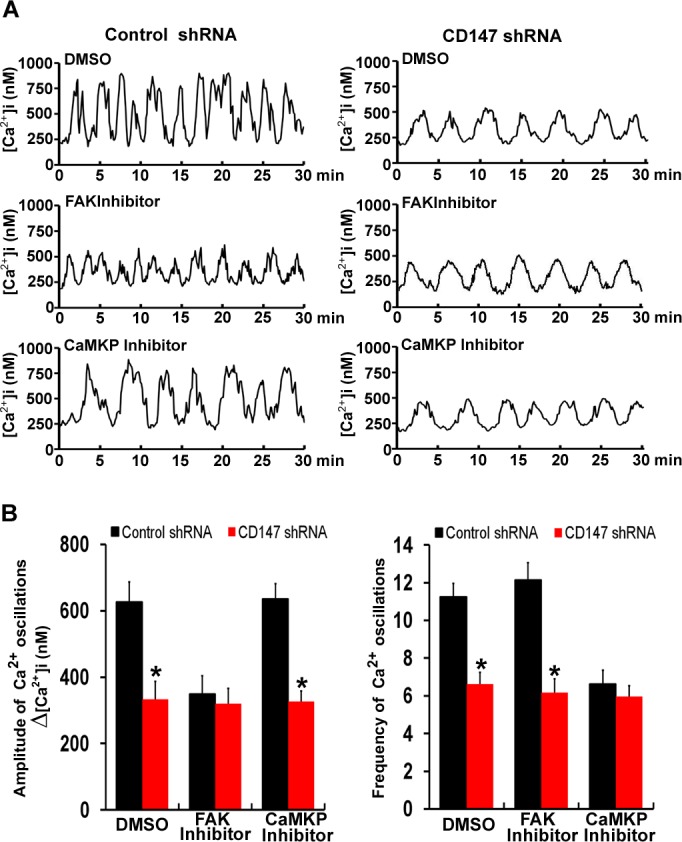
CD147 increases the amplitude of [Ca^2+^]_i_ oscillations through the FAK pathway and increases the frequencies of [Ca^2+^]_i_ oscillations through the CaMKP pathway **A.** Representative traces showing [Ca^2+^]_i_ oscillations in FAK inhibitor-treated cells and CaMKP inhibitor-treated cells after stimulation with EGF were tested. **B.** Histograms of the amplitudes and frequencies [Ca^2+^]_i_ oscillations for FAK inhibitor-treated and CaMKP inhibitor-treated cells after EGF stimulation were shown. Bars represent each sample performed in triplicate, and the error bars represent the standard deviations. *p < 0.05 by Student's *t*-test.

To assess the respective effects of the FAK pathway and CaMKP pathway activated by CD147 on [Ca^2+^]_i_ oscillations, [Ca^2+^]_i_ oscillations were monitored after FAK inhibitor or CaMKP inhibitor treatment. We observed that similar frequencies of [Ca^2+^]_i_ oscillations in both control cells and FAK inhibitor-treated cells. But the amplitudes of the [Ca^2+^]_i_ oscillations were lower in the FAK inhibitor treated cells, and CD147 knockdown did not further decrease the amplitudes of the [Ca^2+^]_i_ oscillations after FAK inhibitor treatment (Figure 6A & 6B). Moreover, similar amplitudes of [Ca^2+^]_i_ oscillations were observed in both control cells and CaMKP inhibitor-treated cells. But the frequencies of the [Ca^2+^]_i_ oscillations were lower in the CaMKP inhibitor-treated cells, and CD147 knockdown did not further decrease the frequencies of the [Ca^2+^]_i_ oscillations after CaMKP inhibitor treatment (Figure 6A & 6B). These results suggested that the FAK signaling pathway is involved in CD147-increased amplitude of the [Ca^2+^]_i_ oscillations and the CaMKP signaling pathway is involved in CD147-increased frequencies of [Ca^2+^]_i_ oscillations.

### Deletion of CD147 inhibites HCC development, thus increasing the survival rate of Alb-Cre; Bsg^fl/fl^ mice by regulating [Ca^2+^]_i_ oscillations

**Figure 7:**
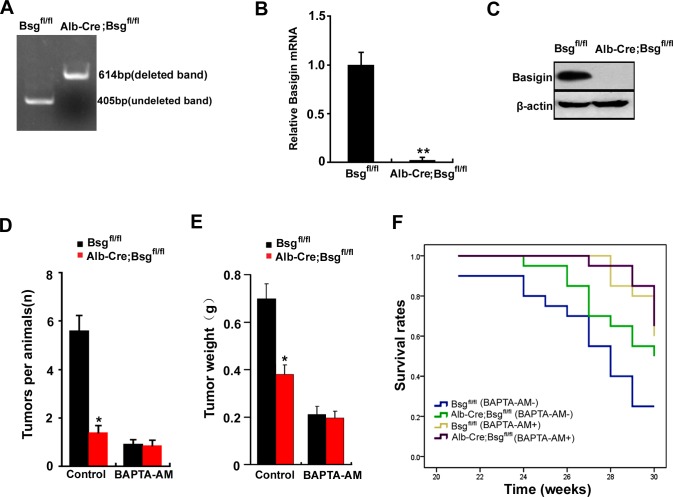
Deletion of CD147 increases the survival rate of Alb-Cre; Bsgfl/fl mice by regulating [Ca^2+^]_i_ oscillations **A.** PCR analysis of the basigin deletion on genomic DNA obtained from the liver. Primers P1, P2 and P3 were added in a same tube for PCR reaction in this experiment. **B.** The relative mRNA level of basigin in liver of Bsg^fl/fl^ mice and ALB-Cre;Bsg^fl/fl^ mice. **, *P* < 0.01. **C.** Western blot analysis of basigin in the liver of Bsg^fl/fl^ mice and ALB-Cre;Bsg^fl/fl^ mice. DEN was used to induce tumors in Bsg^fl/fl^ mice and Alb-Cre; Bsg^fl/fl^ mice. Quantitative analysis data of **D.** the tumor nodule and **E.** the tumor weights were measured. **F.** The survival rate of the mice is illustrated by Kaplan–Meier curves. Six mice per treatment group pooled from three independent experiments are shown. Relevant *P*-values (log-rank test) are depicted for each group. Bars represent each sample performed in triplicate, and the error bars represent the standard deviations. *p < 0.05, **p < 0.01 by Student's *t*-test.

CD147, also named basigin in mice. Mice with conditional null allele for basigin were generated in our lab [24]. To analyze whether CD147-promoted tumor development were mediated by [Ca^2+^]_i_ oscillations *in vivo*, we compared the phenotypes of livers with deletion of basigin. Using the Cre-loxP approach, basigin-floxed mice were crossed with Alb-Cre transgenic mice to derive Alb-Cre; Bsg^fl/fl^ mice as described [24]. We designed PCR primers to amplify the deleted allele (using primers P1 : 5′-CTGGAACTCCTAGCAATC-3′ and P2 : 5′-AGGTGGGTTTTCTGTAAGGT-3′, 614-bp product in length) and undeleted allele (using primers P3 : 5′-CTCTGGGACTCAATGTGTGT-3′ and P2: 5′-AGGTGGGTTTTCTGTAAGGT-3′, 405-bp product in length). The PCR results showed no deleted band in liver obtained from control Bsg^fl/fl^ mice, but a strong deleted band in genomic DNA from liver of the Alb-Cre; Bsg^fl/fl^ mice (Figure 7A). Real-time quantitative PCR and Western blotting showed that basigin was normal in livers of control Bsg^fl/fl^ mice, but was undetectable in liver of the Alb-Cre; Bsg^fl/fl^ mice (Figure 7B & 7C). These results demonstrated that the Bsg^fl^ allele could be specifically deleted in livers in Alb-Cre; Bsg^fl/fl^ mice.

The control Bsg^fl/fl^ and Alb-Cre; Bsg^fl/fl^ mice were injected with DEN. Bsg^fl/fl^ mice and Alb-Cre; Bsg^fl/fl^ mice were both randomized into the two groups: the BAPTA-AM treatment group and the untreated group (Control). The results showed that deletion of basigin markedly reduced tumor weight and the numbers of nodules *in vivo*. But deletion of basigin did not reduce tumor weight and the numbers of nodules following BAPTA-AM treatment (Figure 7D & 7E). We also investigated the survival rate in Bsg^fl/fl^ mice and Alb-Cre; Bsg^fl/fl^ mice with established orthotopic hepatomas after treatment with BAPTA-AM. The results showed that the survival rate were markedly increased in Alb-Cre; Bsg^fl/fl^ mice compared with Bsg^fl/fl^ mice. However, deletion of basigin did not increase the survival rate following BAPTA-AM treatment (Figure 7F). Overall, these results suggest that CD147 promotes HCC development and decreases the survival rate of mice by regulating [Ca^2+^]_i_ oscillations *in vivo*.

## DISCUSSION

**Figure 8:**
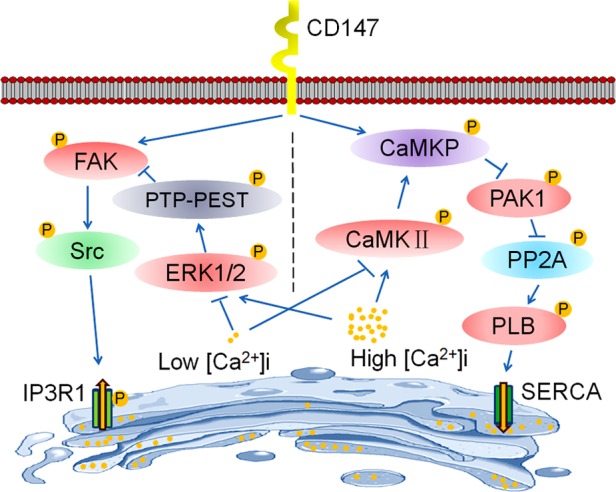
Schematic representation of the major molecular functions of CD147 in the regulation of [Ca^2+^]_i_ oscillations CD147 respectively activates two distinct signaling pathways to regulate [Ca^2+^]_i_ oscillations. On the one hand, CD147 phosphorylates FAK and Src to promote the activation of the IP3R1 channel, which increases Ca^2+^ release from ER stores and enhances the amplitude of [Ca^2+^]_i_ oscillations. On the other hand, CD147 activates CaMKP to inhibit PAK1 phosphorylation and subsequently PP2A activation. CD147-inactivated PP2A enhances phosphorylation of PLB and increases SERCA pump activity, thus accelerating ER Ca^2+^ refilling and enhancing the frequency of [Ca^2+^]_i_ oscillations. Moreover, CD147-promoted ER Ca^2+^ release and refilling are tightly regulated by changing [Ca^2+^]_i_. Under low [Ca^2+^]_i_ conditions, PTP-PEST-mediated FAK dephosphorylation is suppressed, thus facilitating CD147-mediated FAK signaling pathway activation and subsequently the activation of the IP3R1 channel. Meanwhile, low [Ca^2+^]_i_-inactivated CaMKII impairs CD147-induced CaMKP activation, thus blocking the activation of the SERCA pump by CD147. Therefore, CD147 may only induce IP3R1 channel activation under low [Ca^2+^]_i_ conditions. However, under high [Ca^2+^]_i_ conditions, activated CaMKII facilitates CD147-induced CaMKP pathway activation, thus promoting SERCA pump activity. Meanwhile, high [Ca^2+^]_i_-activated ERK1/2 promotes the dephosphorylation of FAK by PTP-PEST, thus antagonizing the phosphorylation of FAK by CD147 and subsequently blocking the activation of the IP3R1 channel by CD147. Therefore, CD147 may only induce SERCA pump activation under high [Ca^2+^]_i_ conditions.

This study highlights the novel mechanism of HCC-associated antigen CD147 in [Ca^2+^]_i_ oscillations regulation of HCC. We discovered that CD147 promotes HCC metastasis and proliferation by enhancing the amplitude and frequency of [Ca^2+^]_i_ oscillation. CD147 respectively activates two distinct signaling pathways to regulate [Ca^2+^]_i_ oscillations. Under low [Ca^2+^]_i_ conditions, CD147 promotes ER Ca^2+^ release by activating FAK-Src pathway dependent IP3R1 channel, thus enhancing the amplitude of [Ca^2+^]_i_ oscillations. Moreover, under high [Ca^2+^]_i_ conditions, CD147 accelerates ER Ca^2+^ refilling by activating CaMKP-PAK1-PP2A-PLB pathway dependent SERCA pump, thus enhancing the frequency of [Ca^2+^]_i_ oscillations (Figure 8). Deletion of CD147 inhibites HCC development, thus increasing the survival rate of Alb-Cre; Bsg^fl/fl^ mice. Therefore, CD147 functions as a critical regulator of ER-dependent [Ca^2+^]_i_ oscillations to promote HCC oncogenicity.

Ca^2+^-mediated signaling pathways have also been shown to play important roles in carcinogenesis such as transformation of normal cells to cancerous cells, tumor formation and growth, angiogenesis, invasion and metastasis [1-4]. Our findings that CD147 regulates [Ca^2+^]_i_ oscillations may account for the various biological functions of CD147 in HCC tumorigenesis.

IP3R1 is an intracellular Ca^2+^-release channel predominantly located on the ER and responsible for a controlled release of Ca^2+^ in the cytoplasm. Functional experiments confirm that tyrosine phosphorylation of IP3R1 leads to a 5-fold increase in affinity for IP3 and promotes IP3R1 open probability. We demonstrated that during ER Ca^2+^ release stage, the higher level of IP3R1 phosphorylation in the presence of CD147, which is mediated by the FAK-Src pathway, would lead to a high level of cytoplasmic calcium following the main Ca^2+^ release from the ER and subsequently a greater level of Ca^2+^ influx, thus enhancing the amplitude of the Ca^2+^ oscillations. Refill of Ca^2+^ into the ER is predominantly mediated by SERCA pump, which is widely distributed within the ER of most cell models. PLB regulates the active transport of Ca^2+^ into the ER via a reversible inhibitory association with the SERCA. Experiments confirm that phosphorylation of PLB at Ser-16 causes relief of SERCA inhibition and permits rapid refill of Ca^2+^ into the ER. We demonstrated that during ER Ca^2+^ refilling stage, CD147 inhibits PAK1-PP2A pathway activity, promoting PLB phosphorylation at Ser16 and dissociation from SERCA, thus enhancing SERCA activity and rapid Ca^2+^ refilling into the ER, therefore increasing the frequency of [Ca^2+^]_i_ oscillations. It is likely that the high expression of CD147 in HCC cells promotes the recycling of the internal Ca^2+^ store, both enhancing Ca^2+^ release and promoting more efficient Ca^2+^ refilling. Thus, differences in the ability of the ER Ca^2+^ stores to recycle Ca^2+^ can account for the apparent differences in the mechanism of CD147-promoted [Ca^2+^]_i_ oscillations in HCC cells.

CD147-induced [Ca^2+^]_i_ oscillations are tightly regulated by [Ca^2+^]_i_. Transient elevations in [Ca^2+^]_i_ have previously been shown to promote FAK dephosphorylation [25]. It has been well documented that Ca^2+^ can modulate the activity of Ras to activate the ERK pathway. The activation of ERK1/2 by high [Ca^2+^]_i_ results in the phosphorylation of PTP-PEST at Ser571, which leads to the recruitment of PIN1 and the cis-trans-isomerization of both FAK and PTP-PEST. The conformational alterations of FAK and PTP-PEST enable them to bind to each other and facilitate subsequent FAK Tyr397 dephosphorylation. We believe that this is the mechanism of FAK activity regulation that balances CD147-induced phosphorylation processes and high [Ca^2+^]_i_-induced dephosphorylation. At the same time, we found that low [Ca^2+^]_i_ inactivated CaMKII, impairs CD147-induced CaMKP activation and blocks the activation of the SERCA pump by CD147, thus preventing further Ca^2+^ refilling of the ER. The preliminary finding that CD147 interacted with CaMKP and enhanced CaMKP activity only in the presence of active CaMKII, which was activated by Ca^2+^, may indicate that the phosphorylation of CaMKP by CaMKII may help CD147 to access its binding site on and thus activate CaMKP [24,25]. These results imply that CD147 is a relevant effector of Ca^2+^ signaling in this system; high [Ca^2+^]_i_ switches CD147 from a Ca^2+^ release enhancer to a Ca^2+^ refill promoter. Under low [Ca^2+^]_i_ conditions, CD147 may only induce IP3R1 channel activation and increase Ca^2+^ release, while under high [Ca^2+^]_i_ conditions, CD147 may only induce SERCA channel activation and the rapid refilling of the ER with Ca^2+^.

In the present study, we discovered that CD147 acts as a critical regulator of ER-dependent [Ca^2+^]_i_ oscillations to promote oncogenic activities and influence the progression of HCC. The inhibition of CD147 may therefore represent a novel approach to inhibit the dysregulation of Ca^2+^ signaling and HCC metastasis and proliferation. In addition to its effects in HCC cells, we have also established that CD147 is highly expressed in other cancers, including breast, lung and bladder cancer gliomas and laryngeal squamous cell, ovarian, renal cell and skin carcinomas [17]. As CD147 is a widely expressed cancer biomarker, its molecular signaling cascade, as well as the mechanism and consequences of its activation in HCC, could also exist in other molecule in developing therapies. In this regard, CD147 may represent a novel anti-cancer target in tumor treatments.

## MATERIALS AND METHODS

### Cell culture

Human HepG2, SMMC-7721, Huh-7, QZG and HL-7702 cells (Institute of Cell Biology, China) were cultured in RPMI 1640 medium (Gibco, Grand Island, USA) supplemented with 10% FBS, 1% penicillin/streptomycin and 2% L-glutamine at 37°C in a humidified atmosphere of 5% CO_2_.

### Cytoplasmic [Ca^2+^]_i_ and ER [Ca^2+^]_i_ measurements

Cells were plated onto coverslips coated with L-polylysine. For Cytoplasmic [Ca^2+^]_i_ measurements, the coverslips were mounted on an imaging chamber and washed with Ca^2+^-free Ringer's solution; the cells were then incubated with Fluo-8/AM (0.1 mg/ml) for 40 min at 37°C. Ca^2+^ signaling was induced by the addition of the indicated reagents. The ER [Ca^2+^]_i_ was measured as described previously [26]. Briefly, cells were loaded by incubation in culture medium containing AM derivatives of Mag-Fura2 for 60 minutes at 37°C and then removed the cytoplasmic fraction of the dye. The cells were subsequently treated with BHQ or Tg to deplete the ER Ca^2+^ stores, and 2mM Ca^2+^ was added to initiate ER Ca^2+^ refilling. [Ca^2+^]_i_ were recorded from Fluo-8/AM or Mag-Fura 2/AM loaded cells, which were excited at 340 nm and 380 nm and imaged with 510 nm filters, and the F340/F380 ratios of the fluorescence intensities were calculated after subtraction of background fluorescence and correction for bleaching. The fluorescence signal was monitored and recorded by an FV300 laser scanning confocal microscope (Olympus, Japan, Tokyo). All experiments were repeated 3 to 6 times. Statistical significance was determined with Student's *t*-test analysis; *p* < 0.05 was considered significant. All data are shown as the average ± SEM.

### Gene silencing

The sense sequence for CD147 shRNA was 5′-GGTTCTTCGTGAGTTCCTC-3′ and negative control shRNA (control shRNA) for CD147 was 5′-GACTTCATAAGGCGCATGC-3′ (Ambion, Austin, TX, USA). The PAK1 siRNA sequence was 5′-TTTCTTCTTAGGATCGCCCACACTC-3′ and negative control siRNA (control siRNA) for PAK1 was 5′- AGTCGACGTCAGCGAAGGC-3′ (Ambion, Austin, TX, USA). The PTP-PEST siRNA sequence was 5′-GGCAATTCCTCAGATATCA-3′ and negative control siRNA (control siRNA) for PTP-PEST was 5′- GGCAATTCCCCAGATATCA-3′ (Ambion, Austin, TX, USA).

### *In vitro* invasion assays

The assay was performed using chambers with polycarbonate filters (8 μm pore size; Millipore). The upper side of a polycarbonate filter was either coated or not coated with Matrigel to form a continuous thin layer. HCC cells (1×10^5^) were resuspended in 300 μL of 0.1% serum medium and added to the upper chamber. The lower chamber was filled with 10% FBS medium (200 μL). After 24 h incubation, the cells on the upper chamber of the filter were removed with a cotton swab, and the cells on the underside were stained and counted.

### Wound healing assay

HCC cells (2×10^6^) were plated in six-well plates and cultured to approximately 90% confluence. The cells were scraped with a pipette tip, washed several times in serum-free medium, and then examined under a phase contrast microscope (Olympus, Japan, Tokyo). The cells were re-fed with 10% FBS medium for 24 h, and images were obtained.

### Cell growth tests

HCC cells were cultured in 96-well plate (1×10^4^ per well) for 24 h. Each condition was performed in triplicate. After incubation, the cells were harvested and counted at every 24 h. The doubling time (T) was calculated according to the formula T = t×log 2/ (log N _t_ - log N _0_). N _t_ indicates the number of cells after culture for t hours, N_0_ indicates the number of cells at the beginning of the culture, and t indicates the culture time.

### Western blot analysis

Cells were lysed in 1% OG buffer (20 mM Tris-HCl, pH 8.0, 150 mM NaCl, 1% OG, 1 mM EDTA, 10 μg/ml leupeptin, 2 μg/ml aprotinin and 1 mM PMSF). A BCA Protein Assay Kit (Pierce Biotechnology, Rockford, Illinois) was then used to determine the total protein concentration, and equal amounts of protein were separated by 10% SDS-PAGE and transferred to a polyvinylidene fluoride (PVDF) microporous membrane (Millipore, Billerica, MA). The membrane was blocked with 5% non-fat milk and then incubated for 2 h at room temperature with the designated antibody. The Western-Light chemiluminescent detection system (Applied Biosystems, Foster, CA) was used for immunodetection.

### Reverse transcription PCR and data analysis

Total RNA was extracted with TRIzol reagent (Invitrogen, California, USA) and reverse transcribed into cDNA with the ReverTra Ace-a kit (Toyobo, Shanghai, China). The primers and probes used were as follows: CD147 forward primer, 5′-TCGCGCTGCTGGGCACC-3′, CD147 reverse primer, 5′-TGGCGCTGTCATTCAAGGA-3′; PTP-PEST forward primer, 5′-GGAGGATGGAGCAAGTGG-3′, PTP-PEST reverse primer, 5′-GCAGCGTGTAACAGGGTT-3′; PAK1 forward primer, 5′-GGGAGAAAGTGAAGCGGTAG-3′, PAK1 reverse primer, 5′-GGTGTCTGGGCAGTTGAG-3′; IP3R1 forward primer, 5′-CTGCTGGCCATCGCACTT-3′, IP3R1 reverse primer 5′-CAGCCGGCAGAAAAACGA-3′; IP3R2 forward primer, 5′-AGCACATTACGGCGAATCCT-3′, IP3R2 reverse primer, 5′-CCTGACAGAGGTCCGTTCACA-3′; IP3R3 forward primer, 5′-CGGAGCGCTTCTTCAAGGT-3′, IP3R3 reverse primer, 5′-TGACAGCGACCGTGGACTT-3′; and GAPDH, forward primer 5′-AATGTCACCGTTGTCCAGTTGC-3′, reverse primer 5′-CACCATCTTAGGAGGAGGAGTAGC-3′. GAPDH mRNA was used as an internal control. The PCR conditions were 1 cycle of 94°C for 5 min; 35 cycles of 94°C for 60 s, 57°C for 30 s, and 72°C for 30 s; and finally 72°C for 5 min. PCR products were electrophoresed on 1% agarose gels.

### Plasmid construction

Full-length IP3R1 (WT) was amplified by PCR. The primers (synthesized by the Shanghai Sangon Co.) were designed as follows: forward primer, 5′- GCTCTAGAGCTGACTACAGAGGAGCAGG-3′ (XbaI) and reverse primer, 5′-AAGGCCTTAACTCATTAGCCATA-3′ (StuI). The Tyr353-mutated IP3R1 (MT) was PCR-amplified using a QuikChange mutagenesis kit (Stratagene, La Jolla, CA, USA) according to the manufacturer's instructions. The tyrosine codon at amino acid 353 in IP3R1 cDNA was changed to a phenylalanine codon. The mutagenic primers used were as follows (mutations shown in bold): forward primer, 5′ - GGACATTCACAAGAGGCTTTCGAGCCATGGTTCT - 3′ and reverse primer, 5′- AGAACCATGGCTCGAAAGCCTCTTGTGAATGTCC - 3′. The products from the full-length IP3R1 (WT) and the Tyr353 mutant IP3R1 (MT) were confirmed by sequencing (Shanghai Sangon, Shanghai, China) and then cloned into pEGFP-N1 (Promega, Madison, WI, USA).

### Co-immunoprecipitation

Cells were washed and resuspended in NPBS and then lysed in 1% OG buffer. A BCA Protein Assay Kit (Pierce Biotechnology, Rockford, IL, USA) was then used to determine the total protein density. Lysates (1 mL) were immunoprecipitated by incubation with 2 μg of the designated antibody and 25 μL of protein A-agarose overnight at 4°C, followed by Western blotting with the designated antibody for 2 h at room temperature. The Western-Light chemiluminescent detection system (Applied Biosystems, Foster City, CA, USA) was used for immunodetection.

### Protein phosphatase assay using MUP as a substrate

The protein phosphatase assays were carried out at 30°C for 20 min in a reaction mixture containing 50 mM Tris–HCl (pH 8.0), 20 mM dithiothreitol, 10 mM MnCl_2_, 0.01% Tween 20, 25 μM Methylumbelliferyl Phosphate (MUP), and 1 μg of CaMKP. The reaction was started by the addition of CaMKP and terminated by the addition of EDTA. MUP would be converted to the soluble fluorescent reaction product methylumbelliferone when they are hydrolyzed by CaMKP. The fluorescence of methylumbelliferone present in the reaction mixture was measured by a spectrofluorometer (Molecular devices, CA, USA) with excitation at 360 nm and emission at 440 nm.

### Animal model

The conditional null allele for basigin in mice were generated in our lab [24]. Alb-Cre transgenic mice were purchased from Model Animal Research Center of Nanjing University. Basigin-floxed mice were crossed with Alb-Cre transgenic mice to generate Alb-Cre;Bsg^fl/fl^ mice (backcrossed to C57BL/6 background for more than eight generations). Male mice were injected intraperitoneally with 25 mg/kg body weight of DEN (Sigma-Aldrich, Munich, Germany) at 14 days of age and then 90 mg/kg body weight of DEN at 6 weeks. Mice were killed at 5 months after birth. All of the animal work was approved by the Animal Care and Use Committee of the Fourth Military Medical University and handled in strict accordance with good animal practice as defined by the relevant national animal welfare bodies.

### Statistical analysis

Statistical analysis was performed using SPSS 13.0 statistical software. The Student's t-test or one-way ANOVA were used to evaluate the statistical significance in the groups. The data are presented as mean ± SD and *p* < 0.05 was considered as significant.

## SUPPLEMENTARY MATERIAL FIGURES


