# Supplementary material for: CD147 reinforces [Ca2+]i oscillations and promotes oncogenic progression in hepatocellular carcinoma
Source: Oncotarget. 2015 Oct 19;6(33):34831–45. doi: 10.18632/oncotarget.5225 (PMC4741493; doi:10.18632/oncotarget.5225)
Supplement: Supplementary file 1 [file oncotarget-06-34831-s001.pdf]

CD147 reinforces $[Ca^{2+}]_i$ oscillations and promotes oncogenic progression in hepatocellular carcinoma

Supplementary Material

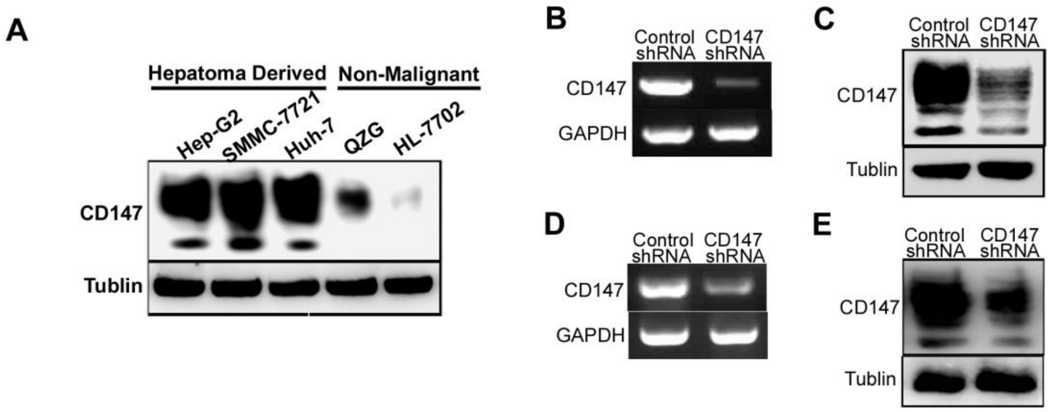

Supplementary Figure 1: CD147 shRNA knockdown CD147 expression in HCC cells. (A) Western blot were performed to examine the CD147 protein levels in a range of hepatoma cancer-derived cells and nonmalignant liver cells. **(B)** RT-PCR and **(C)** Western blot were performed to examine the CD147 mRNA and protein levels in HepG2 cells transduced with shRNA against CD147 using a lentiviral vector. **(D)** RT-PCR and E, Western blot were performed to examine the CD147 mRNA and protein levels of SMMC-7721 cells transduced with shRNA against CD147 using a lentiviral vector.

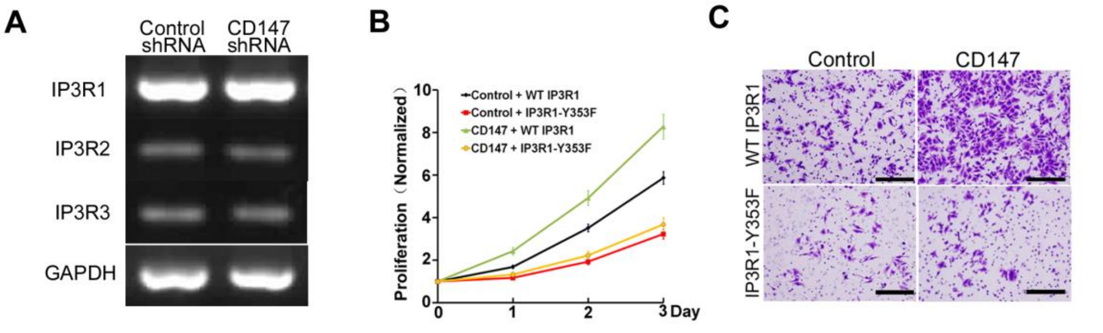

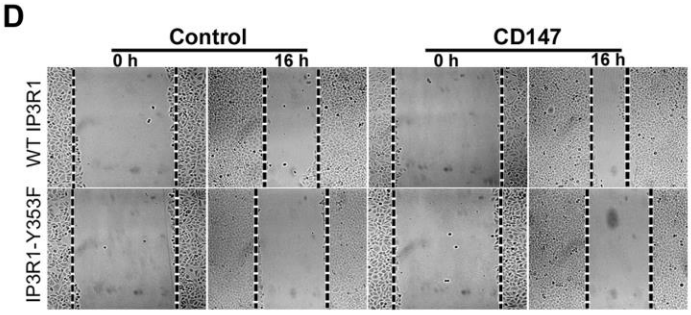

Supplementary Figure 2: RT-PCR was performed to examine the IP3R1，IP3R2 and IP3R3 mRNA levels in control and CD147 knockdown HepG2 cells.

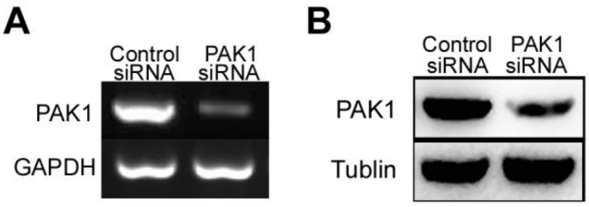

Supplementary Figure 3: PAK1 siRNA knockdown PAK1 expression in HepG2 cells. (A) RT-PCR was performed to examine the PAK1 mRNA levels in HepG2 cells transduced with PAK1 siRNA. **(B)** Western blot was performed to examine the PAK1 protein levels in HepG2 cells transduced with PAK1 siRNA.

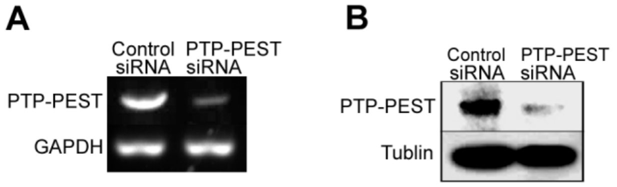

Supplementary Figure 4: PTP-PEST siRNA knockdown PTP-PEST expression in HepG2 cells. (A) RT-PCR and **(B)** Western blot were performed to examine PTP-PEST mRNA and protein levels in HepG2 cells transduced with PTP-PEST siRNA.
